# Alternative splicing diversifies the transcriptome and proteome of the rice blast fungus during host infection

**DOI:** 10.1080/15476286.2022.2043040

**Published:** 2022-03-20

**Authors:** Jongbum Jeon, Ki-Tae Kim, Jaeyoung Choi, Kyeongchae Cheong, Jaeho Ko, Gobong Choi, Hyunjun Lee, Gir-Won Lee, Sook-Young Park, Seongbeom Kim, Sun Tae Kim, Cheol Woo Min, Seogchan Kang, Yong-Hwan Lee

**Affiliations:** a Interdisciplinary Program in Agricultural Genomics, Seoul National University, Seoul, Korea; b Plant Immunity Research Center, Seoul National University, Seoul, Korea; c Korea Bioinformation Center, Korea Research Institute of Bioscience and Biotechnology, Daejeon, Korea; d Department of Agricultural Life Science, Sunchon National University, Suncheon, Korea; e Smart Farm Research Center, Korea Institute of Science and Technology, Gangneung, Korea; f Department of Agricultural Biotechnology, Seoul National University, Seoul, Korea; g NGeneBio Co Ltd, Korea; h Department of Plant Bioscience, Pusan National University, Miryang, Korea; i Life and Energy Convergence Research Institute, Pusan National University, Miryang, Korea; j Department of Plant Pathology and Environmental Microbiology, Pennsylvania State University, University Park, PA USA; k Center for Fungal Genetic Resources, Seoul National University, Seoul, Korea; l Research Institute of Agriculture and Life Sciences, Seoul National University, Seoul, Korea

**Keywords:** Alternative splicing, functional diversification, *magnaporthe oryzae*, pathogenicity, proteome, rice, transcriptome

## Abstract

Alternative splicing (AS) contributes to diversifying and regulating cellular responses to environmental conditions and developmental cues by differentially producing multiple mRNA and protein isoforms from a single gene. Previous studies on AS in pathogenic fungi focused on profiling AS isoforms under a limited number of conditions. We analysed AS profiles in the rice blast fungus *Magnaporthe oryzae*, a global threat to rice production, using high-quality transcriptome data representing its vegetative growth (mycelia) and multiple host infection stages. We identified 4,270 AS isoforms derived from 2,413 genes, including 499 genes presumably regulated by infection-specific AS. AS appears to increase during infection, with 32.7% of the AS isoforms being produced during infection but absent in mycelia. Analysis of the isoforms observed at each infection stage showed that 636 AS isoforms were more abundant than corresponding annotated mRNAs, especially after initial hyphal penetration into host cell. Many such dominant isoforms were predicted to encode regulatory proteins such as transcription factors and phospho-transferases. We also identified the genes encoding distinct proteins via AS and confirmed the translation of some isoforms via a proteomic analysis, suggesting potential AS-mediated neo-functionalization of some genes during infection. Comprehensive profiling of the pattern of genome-wide AS during multiple stages of rice-*M. oryzae* interaction established a foundational resource that will help investigate the role and regulation of AS during rice infection.

## Introduction

Alternative splicing (AS) is one of the regulatory mechanisms for gene expression and produces multiple protein isoforms from a single gene by modulating the maturation of precursor mRNAs (pre-mRNAs) in more than one way to generate heterogeneous transcripts[1]. AS expands the proteome without creating or acquiring new genes [2,3] and serves to modulate protein expression by generating transcripts containing a premature termination codon (PTC). Transcripts with PTC are degraded via the nonsense-mediated decay (NMD) process [4]. AS occurs in the spliceosome, a large ribonucleoprotein complex consisting of small nuclear RNAs (snRNAs) and numerous protein subunits [5]. mRNA isoforms may retain some intron(s) as part of the coding sequence or lack some exon(s) due to the use of different auxiliary splicing element (splicing code), exhibit distinct stability and translational efficiency compared to corresponding annotated mRNAs, and produce proteins with different cellular localization, structure, or function. AS is modulated by members of the SR (Ser-Arg) and heterogeneous nuclear RNP (hnRNP) protein families that recognize the splicing codes [6,7,8,] Expression of SR and hnRNP proteins is affected by environmental conditions, consequently modifying the pattern of splicing under different conditions [8]. Accumulating evidence supports that AS performs crucial tasks. In higher eukaryotes, AS modulates cell differentiation [9], controls response to abiotic and biotic stimuli [8,10], and regulates the circadian rhythm [11]. Almost 95% of the human genes appear to be regulated by AS [12,13]. In *Arabidopsis thaliana*, an RNA-seq analysis showed that 61% of the genes are subjected to AS [14]. Similarly, high-throughput transcriptome analyses revealed that 33–61% of the genes undergo AS in soybean, cotton, maize, and rice [15–19]. Although research on the pattern and role of AS in the kingdom Fungi has been limited [20], available data suggest its significance, including its involvement in pathogenesis [20].

Genome-wide analyses of AS repertoires have been performed in a few fungal species [21–24]. Fungi cause plant and animal diseases and undergo transcriptional reprogramming to evade or compromise host immunity. Human fungal pathogens appeared to increase the extent of AS during infection [25,26]. Three smut fungi (two *Ustilago* species and *Sporisorium reilianum), Colletotrichum graminicola* (a fungus causing corn anthracnose), and *Pseudoperonospora cubensis* (an oomycete that causes downy mildew on cucurbits) generate alternatively spliced transcripts presumably to adapt to their hosts [27,28,29] The AS repertoires observed during plant infection by *Rhizoctonia solani* and *Sclerotinia sclerotiorum* suggested host-specific AS [29,30]. These studies were based on samples collected at a single time point during infection or pooled samples. Because AS is likely subjected to regulation by external conditions and developmental cues, analysing AS patterns at multiple stages of infection is needed to understand how AS is regulated during pathogenesis and which genes undergo AS at specific stages of infection.

In this study, we characterized AS repertoires in the rice blast fungus *Magnaporthe oryzae* at five stages of rice infection and during vegetative growth. This fungus is one of the most economically important plant pathogens [31] by causing up to 30% loss of the rice produced globally [32]. The fungus is hemibiotrophic, switching its lifestyle from biotrophy to necrotrophy during infection. Genome sequences of strains isolated from diverse rice varieties and other grass species, transcriptomes under *in vitro* and *in planta* conditions, and molecular research tools are available [33], making *M. oryzae* an excellent model for studying fungal pathogenesis mechanisms. Several studies of *M. oryzae* suggested the importance of AS for pathogenesis. The proteins encoded by the *RBP35, MoGrp1* [34,35], and methylation regulator (*MoHMT1*) genes [36] interact with spliceosome components. Reduced virulence of the deletion mutants of these genes suggested the importance of proper splicing regulation for pathogenesis. Two *M. oryzae* genes subjected to AS, including *MoYPD* and *MoSOM1*, contribute to virulence [37,38]. Two AS isoforms derived from *MoPTEN*, a gene important for growth and pathogenesis, seem to perform distinct functions [39]. A previous study investigated AS profiles in an *M. oryzae* (previously called *M. grisea*) strain by sequencing expressed sequence tags (ESTs) [40]. However, because this study did not include ESTs derived from infected plants, a comprehensive AS profiling of *M. oryzae* during the disease cycle is needed to uncover the full extent and variation of AS during host infection and proliferation.

We previously reported high-quality RNA-seq data from mycelia (vegetative tissue) of *M. oryzae* and the samples collected at the following stages of infection: pre-penetration (18 hpi), biotrophy (27 hpi and 36 hpi), and necrotrophy (45 hpi and 72 hpi) [41]. We reanalysed this data set to identify the *M. oryzae* genes subjected to AS and the type, abundance, and infection stage-specific pattern of mRNA isoforms. Translation of some isoforms was confirmed via a proteomic analysis. We applied the concept of isoform abundance to evaluate whether transcript functionality likely changes under different conditions. Neo-functionalization caused by AS-mediated changes in protein domains seems to occur during infection, suggesting the likely involvement of AS-mediated transcriptome remodelling in pathogenesis.

## RESULTS

### *Identification of the* M. oryzae *genes containing multiple exons*

We published RNA-seq datasets derived from KJ201, an *M. oryzae* field isolate from diseased rice, during infection [41]. However, because we used the genome of 70–15, a laboratory strain created through a series of genetic crosses [42], as a reference for mapping transcript sequences of KJ201, we could not align some KJ201 reads due to their genome differences. To support a more accurate transcriptome analysis of KJ201, we sequenced the genome of KJ201. The assembled genome, represented by 123 scaffolds with a total length of 41.7 Mb and N50 values of 2.3 Mb, contains 13,306 genes (Table 1). Synteny comparison revealed that the genomes of KJ201 and 70–15 are mostly conserved with a few segmental differences (Supplementary Figure S1A). However, only 9,951 KJ201 genes (78.6%) have orthologs in 70–15 when their predicted proteomes were compared using >90% sequence identity and coverage as the threshold. Moreover, only 62.5% of the KJ201 genes showed the same number of intron(s) in their 70–15 orthologs (Supplementary Figure S1B). Based on the annotated gene model of KJ201, 10,178 (76.5%) genes are predicted to contain multiple exons (Supplementary Table S1).

**Table 1.** Statistics of KJ201 genome

Genome statistics	KJ201
Genome size (Mb)	41.7
N50 (bp)	2,318,557
L50	14
No. of scaffolds	123
No. of protein-coding genes	13,306

### Analysis of AS patterns in KJ201 during vegetative growth and infection

**Figure 1.**
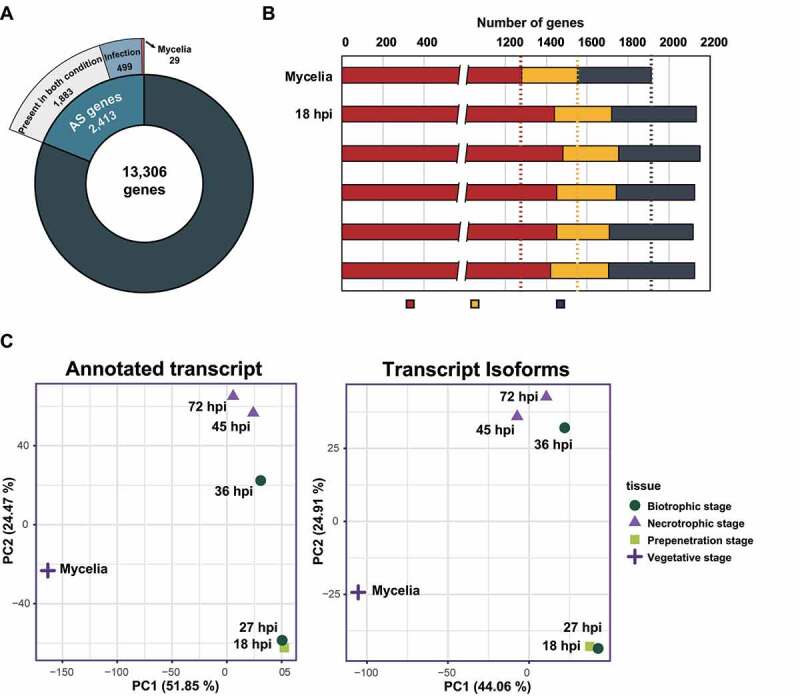
Characteristics of AS in *M. oryzae* KJ201 under different conditions.

We analysed AS profiles in KJ201 using our published RNA-seq data [41] with the KJ201 genome as the reference (Table 2). Transcripts potentially generated via AS were identified using a two-step pipeline (Supplementary Figure S2) [43]. We initially identified 2,772 candidate genes with their transcripts undergoing AS under one or more conditions. After filtering out those expressed at very low levels and corresponding to chimeric transcripts, we found 4,270 novel mRNA isoforms transcribed from 2,413 genes (18.1% of the total genes) (Fig. 1A; Supplementary Table S2). In total, 1,914 genes produced AS isoforms in mycelia (Fig. 1B). More genes were subjected to AS during infection (Fig. 1B): 2,135 genes at 18 hpi (the stage of appressorium formation), 2,153 and 2,127 genes at 27 and 36 hpi, respectively (biotrophic stage), and 2,118 and 2,127 genes at 45 and 72 hpi, respectively (necrotrophic stage). A Principal Component Analysis (PCA) of the annotated transcripts and their isoforms (Fig. 1C) showed three distinct clusters. Both types of transcripts present in mycelia were distinct from those observed in infected rice (Fig. 1C).

**Table 2.** Statistics of KJ201 transcripts analysed

	Mycelia	18 hpi	27 hpi	36 hpi	45 hpi	72 hpi
Proportion of fungal reads (%)	93.33	6.83	4.08	4.41	7.43	25.65
No. of mapped reads (x1,000)	40,608	15,763	8,378	9,441	16,565	53,444
Depth (X)	204.6	90.5	54.2	60.9	96.2	247.8

(a) Among 13,306 annotated genes of KJ201, 2,413 are subjected to AS under one or more conditions. Transcript isoforms from 29 and 499 genes were detected only in mycelia and during infection, respectively. The remaining 1,883 genes produced AS transcripts under both conditions. (b) The number of AS genes in each sample predicted using three FPKM values for cut-off. (c) PCA analysis of the annotated transcripts and their isoforms. The log2 FPKM values of individual transcripts within each sample are used to calculate PCA distances. The standard PCA function implemented in prcomp was used for this analysis.

### Vegetative- and infection-specific AS isoforms and expression patterns of putative AS regulators

**Figure 2.**
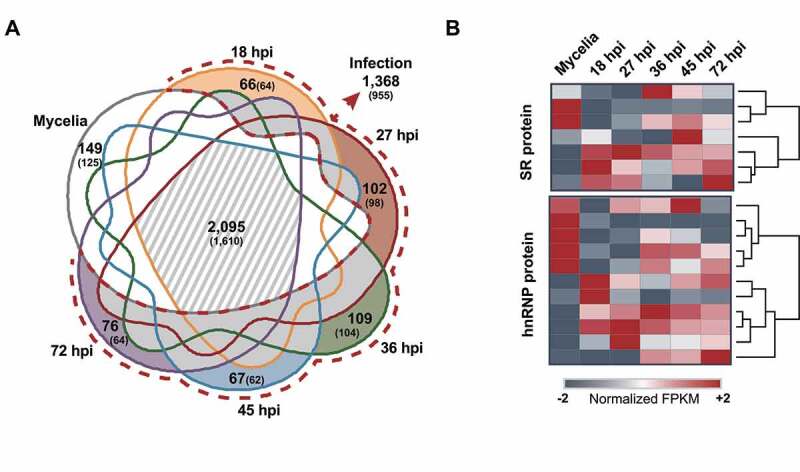
AS repertoires and the expression patterns of putative AS regulator genes under different conditions.

The AS isoforms detected under different conditions were compared to determine how their production was regulated (Fig. 2A). Among the 4,270 isoforms identified, 2,095 (49.1%) isoforms, produced from 1,610 genes, were present under all conditions. The total number of isoforms produced during infection was 1,368 (32.7%) from 955 genes, with 696 isoforms (16.3%) from 499 genes being produced only during infection. The number of vegetative stage-specific isoforms was 149 (125 genes). The highest numbers of infection-specific isoforms were detected during the biotrophic stage (109 (104 genes) and 102 (98 genes) isoforms at 27 and 36 hpi, respectively).

(a) Venn diagram showing the number of AS isoforms and genes (in parenthesis) at each stage. The light grey dashed region denotes the AS isoforms produced in mycelia as well as during infection (2,095). Each stage is colour-coded: mycelia (grey line), pre-penetration (yellow line), biotrophic (red and green line), and necrotrophic (blue and purple line). The stage-specific isoforms are colour coded. The AS isoforms detected only during infection are shown in a dashed red line. (b) Heatmap showing how the SR and hnRNP protein-coding genes are expressed under these conditions.

We analysed expression patterns of the 81 genes encoding various components of the spliceosome (Supplementary Figure S3). They were differentially expressed under these conditions, with some being induced in mycelia while others expressing higher during infection. We also analysed how the genes encoding putative AS regulators, including seven SR and 11 hnRNP proteins, are transcribed and processed under the same conditions to determine if AS regulates their expression. Ten and six genes were up- and down-regulated, respectively, at one or more infection stages (Fig. 2B). Five AS regulator genes produced isoforms, with the isoforms of three genes displaying expression patterns different from their annotated forms (Supplementary Figure S4).

**Figure 3.**
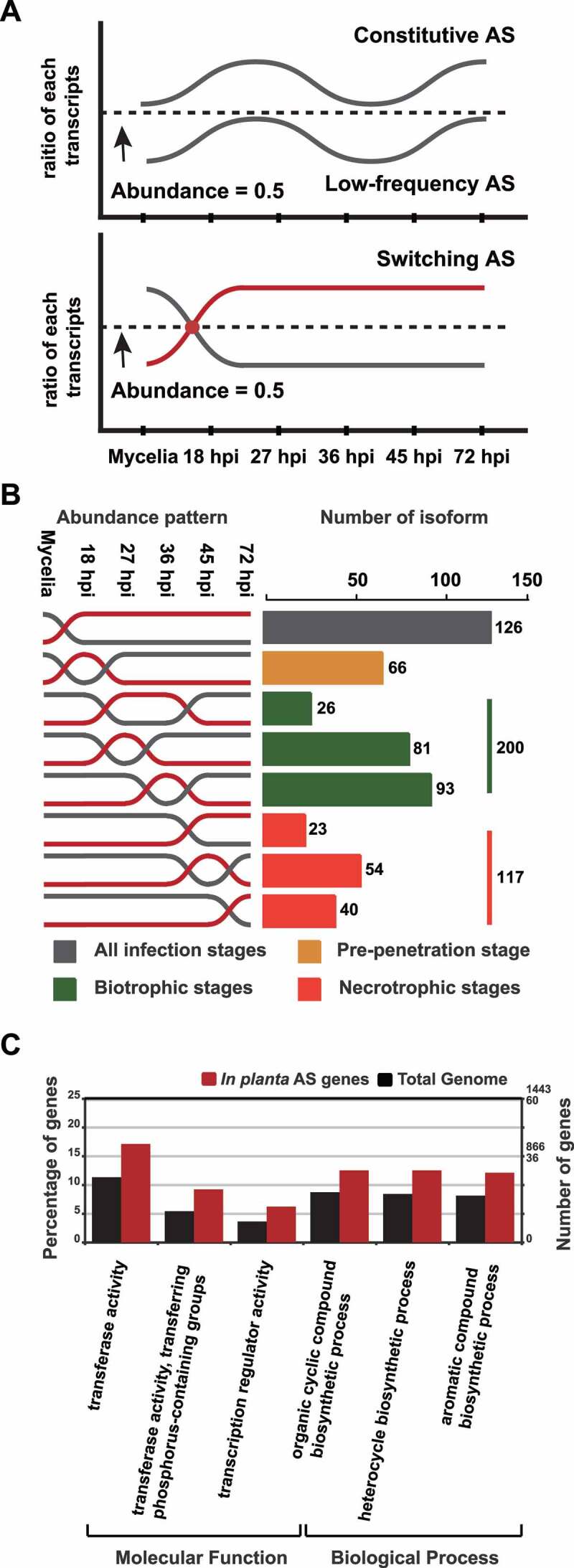
Three types of AS isoforms produced under different conditions and characteristics of Switching AS-type isoforms.

Relative expression levels of AS isoforms at each stage were analysed (Supplementary Figure S5). We applied the concept of transcript usage [44], which indicates the relative abundance of individual isoforms compared to corresponding annotated mRNAs in each sample, to predict which genes and isoforms are likely important for infection. Their relative abundance was calculated using the following formula: Fragment Per Kilobase of transcript per Million mapped reads (FPKM) of isoform/(FPKM of isoform + FPKM of annotated form). We group them into three clusters based on their relative abundance pattern: (1) Constitutive AS (relative abundance of 0.5–1 under all conditions), (2) Low-frequency AS (relative abundance of < 0.5 under all conditions), and (3) Switching AS (those fluctuating between 0.5–1 and <0.5 under different conditions) (Fig. 3A). We found that 658 isoforms (559 genes) belong to Constitutive AS. Switching AS included 868 isoforms (636 genes) and was more frequently observed during infection than in mycelia. Low-frequency AS included 1,949 isoforms (1,260 genes). For Switching AS, 126 isoforms (109 genes) were abundant throughout all infection stages. During infection, 200 isoforms (179 genes) at the biotrophic stage (27–36 hpi), 117 isoforms (108 genes) at the necrotrophic stage (45–72 hpi), and 66 isoforms (65 genes) at the pre-penetration stage (18 hpi) were identified (Fig. 3B; Supplementary Table S3).

(a) The diagrams show the relative abundance (the ratio of isoforms over annotated forms) of three types of AS transcripts during the growth stages analysed. Constitutive AS and Low-frequency AS isoforms indicate those with the relative abundances of > 0.5 and < 0.5, respectively. Switching AS-type isoforms include those fluctuating between the first two types between mycelia and infected rice. (b) Expression patterns of the following four groups of Switching AS-type isoforms during infection: grey box (the isoforms abundantly produced at all infection stages), yellow box (those produced higher than corresponding annotated forms only at the pre-penetration stage, 18 hpi), green box (those produced higher than corresponding annotated forms only at the biotrophic stage, 25 hpi or 36 hpi), and orange box (those produced higher than corresponding annotated forms only at the necrotrophic stage, 45 hpi or 72 hpi). (c) The GO terms enriched among the genes that produce Switching AS-type isoforms (P-value < 0.05).

Gene Ontology (GO) enrichment analysis was performed to identify likely functions of the genes belonging to Switching AS. Most of them are predicted to be involved in heterocyclic compound binding, ion binding, or hydrolase activity (Supplementary Table S4). Significantly enriched GO terms compared to the whole gene set included phospho-transferase, transcription regulator activities, and cyclic compound biosynthetic processes (Fig. 3C). We further investigated the GO term-associated patterns at each stage. Only the necrotrophic stage showed enriched terms (heterocyclic compound and ion bindings) (Supplementary Table S5). We analysed the composition of those associated with infection-enriched phospho-transferases and transcription regulator activities. We found that 70 kinase genes produced AS isoforms. Among them, 20 generated Switching AS-type isoforms (Supplementary Figure S6A), but no subfamilies were enriched in this group compared to the total kinases (Supplementary Figure S6B). Among 139 predicted TF genes that produced AS isoforms (Supplementary Figure S7A), C_2_H_2_ zinc finger (28.1%, *p* = 0.04415) and High Mobility Group (HMG; 18.8%, *p* = 0.04427) were enriched among 35 Switching AS-type isoforms (Supplementary Figure S7B). We checked whether the proteins encoded by those belonging to Switching AS likely participate in pathogenesis by matching them to the pathogenicity-related genes curated in PHI-base [45]. Homologs of 39 genes have been functionally studied (Supplementary Table S6). Among them, 22 genes, including oxidative stress-related (*ABC1, RanBP, TIG1, MoARK1*) and autophagy-related (*MoAtg1, MoAtg2*), have been shown to be involved in pathogenesis.

### Intron retention is the most common type of AS

**Figure 4.**
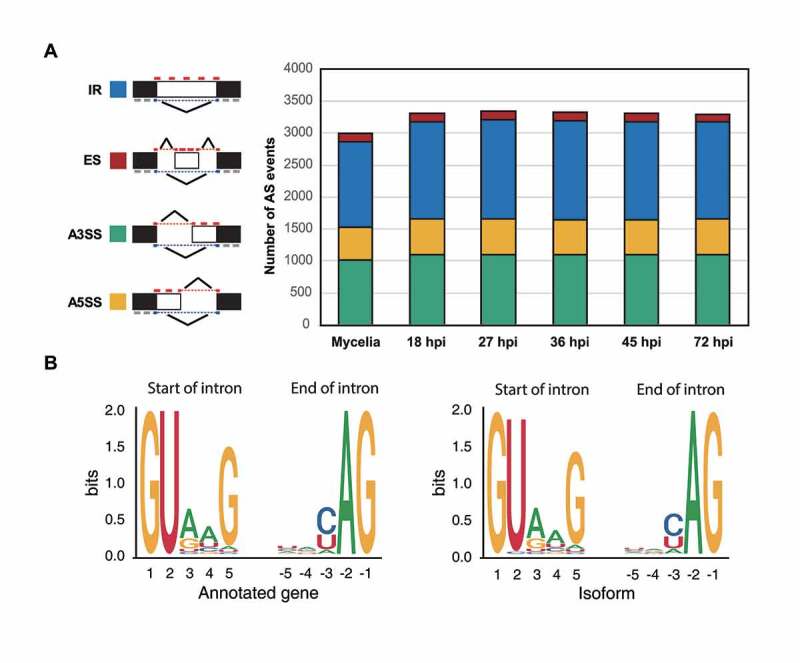
Four types of AS observed and their distribution patterns under different conditions.

The number of AS isoforms produced from 2,413 genes ranged from one to 10, with 1,365 genes expressing only one isoform (Supplementary Table S7). Intron retention (IR) was the dominant type under all conditions (Fig. 4A). Those generated via alternative donor (A3SS), alternative acceptor (A5SS), and exon skipping (ES) were also observed. We compared intron splicing sites between annotated mRNAs and their isoforms. In annotated mRNAs, 6,362 introns have the canonical splicing site GT|AG, and only two introns have a non-canonical splicing site (GC|AG) (Fig. 4B). In contrast, 269 introns among the isoforms have GC|AG.

(a) Four types of AS, including intron retention (IR), exon skipping (ES), alternative 3’ splicing site (A3SS), and Alternative 5’ splicing site (A5SS), observed at the vegetative and five infection stages are shown. The black box denotes exon. (b) Sequence patterns at the splicing sites in annotated genes (left) and isoforms (right). The LOGO diagrams show 5 bp downstream and upstream from the splicing junctions.

### Predicted changes in protein domain structure caused by AS

**Figure 5.**
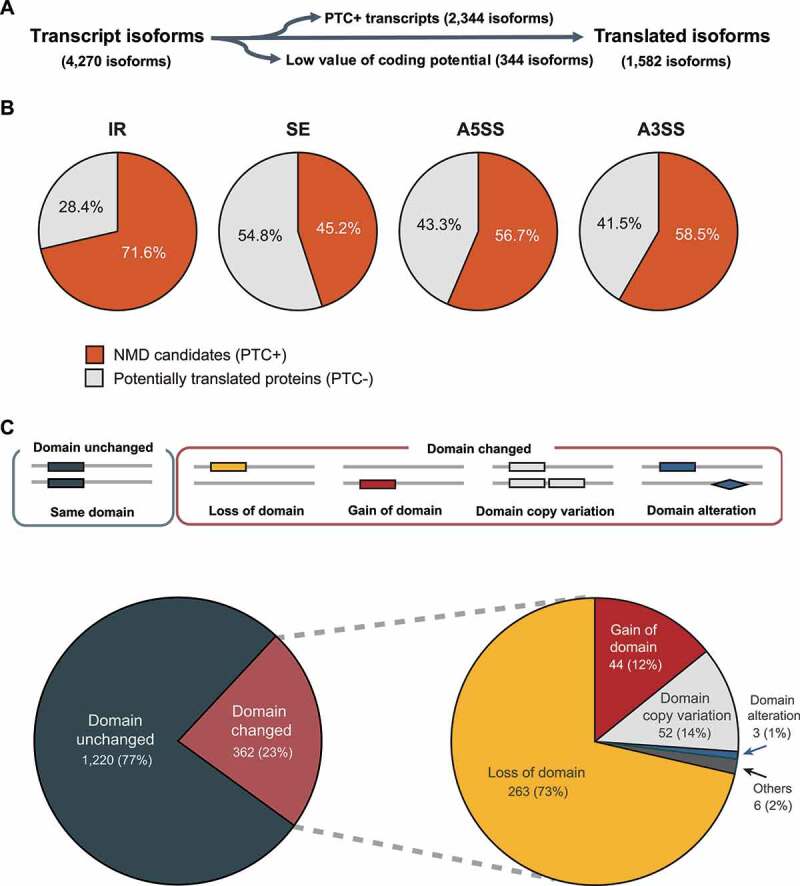
Domain variation patterns observed among the translated isoforms.

The protein-coding ability of individual AS isoforms was evaluated by checking coding potential and Premature Termination Codon (PTC) (Fig. 5A). The coding potential was used to identify potentially non-coding transcripts. Among the 4,270 isoforms examined, 3,926 (91.9%) display protein-coding potential. Some mRNAs produced via AS are unlikely to be translated because of nonsense mediated decay (NMD). Among the isoforms with coding potential, 2,344 had PTC. Whereas 71.6% of PTC were generated via IR, less than 60% of the other splicing patterns (exon skipping, alternative 3’ or 5’ splicing site) created PTC (Fig. 5B). Gene ontology analysis according to the presence or absence of PTC (PTC+ or PTC-) showed differences in functional categories. Five enriched terms were associated with the genes in PTC+, whereas 18 terms were enriched in those belonging to PTC- (Supplementary Table S8). The remaining 1,582 isoforms did not have PTC and were expected to be translated. (Supplementary Table S9). Among the merged set of *ab initio* protein predictions, 1,220 (77%) isoforms could be translated without Pfam domain change. However, 368 (22%) isoforms were predicted to encode proteins with altered Pfam domain(s) (Fig. 5C) and could be categorized into multiple types. The most frequent one was the loss of domain(s) (263 isoforms). Domain copy variation (52 isoforms), gain of domain (44 isoforms), and domain alteration (3 isoforms) were also observed (Fig. 5C).

(a) A filtration pipeline used to remove non-translated isoforms and isoforms with low coding potential. (b) Proportions of NMD candidates (PTC+) in four major isoform types: Intron retention (IR), ES (Exon skipping), A3SS (Alternative 3’ splicing site), and A5SS (Alternative 5’ splicing site) (c) The pie graphs show the distribution patterns of domain change patterns among translated AS transcripts.

### Structural changes caused by AS among secreted proteins

**Figure 6.**
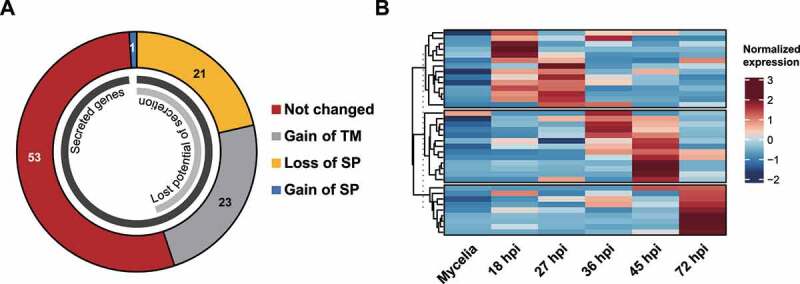
Predicted modifications of secreted proteins due to AS.

Pathogenic fungi secrete diverse proteins as virulence factors. We assessed whether AS affects the production of secreted proteins. The genome of KJ201 contains 1,340 genes predicted to encode secreted proteins. Among 168 genes that produced AS isoforms, those derived from 71 genes (42.3%) did not have PTC. The sequences of signal peptide/membrane anchor domains in 44 proteins seem to be affected by AS (Fig. 6A), with 43 isoforms being predicted to produce proteins without a signal peptide and one gaining a signal peptide. Additionally, the proteins produced from 23 isoforms were predicted to gain a transmembrane motif without any change in their signal peptide. Twenty-eight genes encoding small secreted proteins, candidate effectors, produced AS isoforms at specific stages of infection (Fig. 6B).

(A) Three types of changes that potentially affect protein secretion are shown. Two types, loss of signal peptide (SP) and gain of transmembrane motifs (TM), are expected to disrupt protein secretion. A gain of SP was also observed. (B) The expression pattern of AS isoforms of secreted protein-coding genes.

### Validation of the production and translation of some AS isoforms

**Figure 7.**
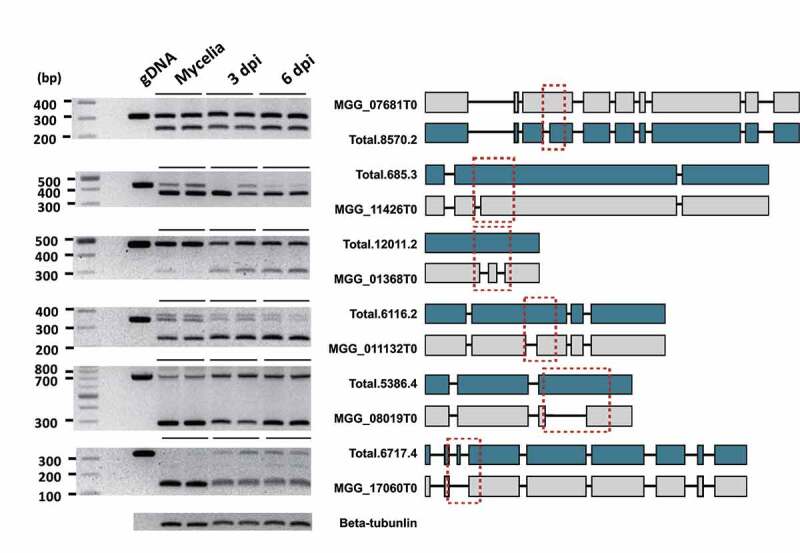
Validation of the transcripts produced via AS and the resulting structural changes.

We used RNA samples from mycelia and infected rice leaves (3 dpi and 6 dpi) to validate the production of predicted mRNA isoforms for 10 genes. Both annotated and isoforms were detected via RT-PCR for six genes (Fig. 7): intron retention in the isoforms of five genes (orthologs of MGG_07681T0, MGG_11426T0, MGG_01368T0, MGG_011132T0, and MGG_08019T0) and exon skipping in one gene (MGG_17060T0). We also conducted a label-free quantitative mass spectrometry to confirm the translation of some isoforms. We identified the proteins encoded by 266 genes in the proteome data. As expected, most of the proteins were produced from annotated mRNAs. However, 24 proteins were translated from isoforms, with 16 protein isoforms being produced during infection (Table 3)

**Table 3.** List of the proteins produced using transcript isoforms generated via AS

Protein encoding transcript variants		Functional annotation^++^
Annotation*	Detection^+^	
maker-scaffold000002-augustus-gene-36.61-mRNA-1	Infection (7 dpi), Mock (0 dpi)	Chitin recognition protein
maker-scaffold000077-augustus-gene-2.104-mRNA-1	Infection (7 dpi), Mock (0 dpi)	RNA-dependent RNA polymerase
augustus-scaffold000002-processed-gene-19.59-mRNA-1	Infection (7 dpi), Mock (0 dpi)	U6 snRNA phosphodiesterase
maker-scaffold000097-snap-gene-0.69-mRNA-1	Infection (7 dpi), Mock (0 dpi)	-
maker-scaffold000097-augustus-gene-1.54-mRNA-1	Infection (7 dpi), Mock (0 dpi)	-
maker-scaffold000098-augustus-gene-0.137-mRNA-1	Infection (7 dpi), Mock (0 dpi)	-
augustus-scaffold000110-processed-gene-1.149-mRNA-1	Infection (7 dpi), Mock (0 dpi)	Ribosome-associated complex head domain
snap-scaffold000002-processed-gene-37.21-mRNA-1	Infection (7 dpi), Mock (0 dpi)	-
augustus-scaffold000002-processed-gene-41.0-mRNA-1	Infection (7 dpi), Mock (0 dpi)	Ubiquitin system component Cue
maker-scaffold000032-snap-gene-14.129-mRNA-1	Infection (7 dpi), Mock (0 dpi)	Helicase
augustus-scaffold000061-processed-gene-9.89-mRNA-1	Infection (7 dpi), Mock (0 dpi)	Histidyl-tRNA synthetase
maker-scaffold000002-snap-gene-7.10-mRNA-1	Infection (7 dpi), Mock (0 dpi)	Tetrahydrofolate dehydrogenase
maker-scaffold000031-snap-gene-38.67-mRNA-1	Infection (7 dpi), Mock (0 dpi)	Ras family
maker-scaffold000003-augustus-gene-2.238-mRNA-1	Infection (7 dpi)	-
maker-scaffold000095-augustus-gene-8.79-mRNA-1	Infection (7 dpi)	Ras family
maker-scaffold000096-snap-gene-1.11-mRNA-1	Infection (7 dpi)	Oxysterol-binding protein
maker-scaffold000110-augustus-gene-1.52-mRNA-1	Mock (0 dpi)	-
maker-scaffold000002-augustus-gene-31.44-mRNA-1	Mock (0 dpi)	G-protein alpha subunit 14
maker-scaffold000011-snap-gene-4.92-mRNA-1	Mock (0 dpi)	Peroxidase
maker-scaffold000062-augustus-gene-30.7-mRNA-1	Mock (0 dpi)	UAA transporter family
maker-scaffold000060-augustus-gene-13.71-mRNA-1	Mock (0 dpi)	Calreticulin/calnexin
maker-scaffold000095-snap-gene-7.11-mRNA-1*	Mock (0 dpi)	-
maker-scaffold000002-augustus-gene-31.42-mRNA-1*	Mock (0 dpi)	HECT-like Ub-conjugating enzyme (E2)-binding
maker-scaffold000092-augustus-gene-0.5-mRNA-1*	Mock (0 dpi)	Common central domain of tyrosinase 65

* The protein sequence was determined using the longest ORF prediction

**^+^** The condition(s) in which each isoform was detected.

**^++^** Predicted function of the resulting protein.

AS of selected genes was validated using RT-PCR. The samples analysed are mycelia and rice leaves collected at 3 days post-inoculation (dpi) and 6 dpi. Genomic DNA was used as the control for RT-PCR reactions. The structural differences between the annotated form (grey) and isoform (blue) are shown. The red boxes indicate the amplified regions of DNA and cDNA.

## Discussion

AS occurs widely in eukaryotes and expands the transcriptomic and proteomic diversity without increasing the number of genes. AS is known to be involved in regulating disease resistance in plants [46]. Fungal transcriptome can also be diversified via AS during host invasion [26]. In this study, we analysed the type and temporal production pattern of AS isoforms in *M. oryzae* during rice infection to investigate whether AS potentially contributes to pathogenesis. Instead of using 70–15, a widely employed laboratory strain, we used KJ201, a field strain, for this analysis. The RNA-seq data used were highly enriched for fungal reads compared to the transcriptome data derived from plants infected with other *M. oryzae* isolates (Supplementary Table S10), helping analyse AS profiles during infection. We sequenced the genome of KJ201 to increase the accuracy of analysing AS isoforms at different stages of infection, resulting in a relatively high mapping rate compared to that using 70–15 (KJ201: 150 M reads vs. 70–15: 142 M reads).

A previous study found AS isoforms produced by 134 genes in 70–15 [40]. However, because only >28,000 ESTs were sequenced, this analysis could not fully reveal the global pattern of AS. Besides, this study did not include ESTs corresponding to *in planta* expressed *M. oryzae* genes. We used high-depth RNA-seq data (Table 2) and a two-step AS profiling pipeline to identify and quantify AS isoforms produced at multiple stages of infection (Supplementary Figure S2). Unlike earlier studies with other fungal pathogens [29–32] which were performed using samples collected at a single time point or mixed samples, samples representing multiple infection stage were analysed in our analysis, leading to the identification of 4,270 novel isoforms derived from 2,413 genes. Among the total isoforms, 696 isoforms transcribed from 499 genes were only produced during infection (Fig. 1), suggesting that some such AS isoforms could function in colonizing host cells. Analyses of other developmental stages and stress conditions will likely increase the number of genes subjected to AS.

Plants undergo AS during pathogenic and symbiotic microbial interactions [47]. Plant AS repertoire has been shown to be reprogrammed by fungal and oomycete infections [48], suggesting that both plants and pathogens employ AS to modulate responses to biotic stimuli. During infection, the extent of AS in *M. oryzae* appears to increase, and the production of AS isoforms is regulated differentially depending on the stage of infection (Fig. 2). The low depth of reads corresponding to *M. oryzae* transcripts during the biotrophic stage (27 and 36 hpi) likely led to underestimating the extent of AS [49]. However, detection of more genes subjected to AS during this stage than the vegetative stage suggested more active AS occurrence during infection (Fig. 3B). During the biotrophic stage, the collective outcome of many molecular interactions between rice and *M. oryzae* determines whether infection can progress or not. The diversification of AS repertoire in *M. oryzae* during infection may represent the pathogen’s attempt to overcome host defence systems.

To assess the potential role of AS during infection and identify pathogenesis-associated genes regulated by AS, we queried PHI-base [45] using the genes subjected to AS. The genes for two autophagy-related proteins and four oxidative stress regulators in *M. oryzae* were subjected to infection-specific AS. The anti-oxidation defence mechanism in *M. oryzae* confers tolerance to host oxidative burst [50]. Autophagy is a strategy used to adapt to low nutrition environment during the early stage of infection [51]. These findings suggest the involvement of AS in modulating stress response. The genes subjected to AS are enriched for TFs and phospho-transferases (Fig. 3C, Supplementary Table S4). The human fungal pathogen *Candida albicans* utilizes TFs and kinases (one of the most well-characterized groups of phospho-transferases) to respond to environmental conditions including manage various stresses [52]. Considering the well-established role of TFs and phospho-transferases in regulating infection processes [53], the observed regulation of their transcription via AS supports the role of AS in pathogenesis. Three Switching AS-type genes related to virulence (*CHS7, MoBIR1*, and *MGG*_*03451*), identified via the search of PHI-base, may produce proteins with functional domain alteration via AS, suggesting that AS could affect the translation of virulence genes (plant cell wall degrading enzymes) and host adaptation genes (autophagy). Functional studies of the AS isoforms derived from these genes will be necessary to validate their involvement in infection.

AS is controlled by regulatory factors such as SR proteins and *trans*-acting hnRNP proteins [6]. It was proposed that the complexity of AS correlates with the number of these regulatory factors [54. Relative to seven genes encoding SR proteins in *M. oryzae, Saccharomyces cerevisiae* has only one, and *Schizosaccharomyces pombe* carries two [25]. These regulatory factors are known to function in tissue and stress-specific manners [55]. For example, two *Fusarium graminearum* SR proteins, FgSrp1 and FgSrp2, modulate AS differently. FgSrp1 is involved in pathogenesis, whereas FgSrp2 is only involved in vegetative growth [56,57]. The genes encoding splicing regulatory factors were expressed differently at the vegetative and infection stages (Fig. 2B), which is consistent with distinct AS patterns observed (Supple Figure S4). Phenotypes of mutants in putative splicing genes showed that two genes could specifically affect pathogenicity (Supplementary Table S11).

The most predominant AS mechanism in *M. oryzae* was intron retention (IR), and exon skipping did not occur frequently (Fig. 4A). This pattern was observed in other fungi and plants [58,59]. IR prevalently generates mRNAs harbouring PTC which are subjected to decay pathway [14,60]. Three-fourth of the isoforms generated via IR contained PTC, but the proportions of PTC-containing isoforms via other types of AS were lower than that caused by IR (Fig. 5B).

AS isoforms are known to cause NMD or increase the proteome complexity. Production of alternative proteins created via AS was observed in *Lachancea kluyveri* and *P. cubensis* [61,62]. The genes producing isoforms harbouring PTC were present across all functions, whereas the genes producing isoforms without PTC were enriched with GO associated with regulatory roles (Supplementary Table S8). Our *in silico* analysis of the domains of predicted proteins without PTC (Fig. 5) indicated that functional changes via AS may occur. A label-free quantitative mass spectrometry analysis showed that 24 proteins, including 16 proteins only expressed during infection, were translated from the isoforms, confirming the production of heterogeneous proteins from a single gene (Table 3) and suggesting that such changes are likely associated with host infection.

Previous studies revealed that AS could regulate virulence. We could detect multiple AS transcripts of *MoSOM1*, a gene regulating infection-related morphogenesis [38] *in planta*, suggesting that AS could regulate some pathogenesis-related genes. We also found AS events derived from *MoPTEN*, a gene that was shown to produce two transcripts, in our RNA-seq data. One isoform (*MoPTEN-1*) is involved in conidiation and appressorial formation while the second (*MoPTEN-2*) is needed for invasive hyphal growth [39], suggesting that motif changes caused by AS made them perform different functions.

Secreted pathogen proteins play diverse critical roles, including the modification of host-associated environments and defence processes, during infection [63]. Since modification of the signal peptide and transmembrane motifs via AS could alter their secretion, AS can potentially serve as one of the regulatory mechanisms for their function. This type of motif alteration of a small secreted protein by AS was previously reported in cucurbit downy mildew fungus *P. cubensis* [62]. In *M. oryzae*, 28 small secreted protein-encoding genes, including the gene encoding the effector BAS3 [64], produced mRNA isoforms, supporting that AS may play important roles in infection by modulating the production of effectors during host invasion. In summary, our study revealed the comprehensive temporal pattern of AS while *M. oryzae* infects rice, which will help understand the AS mechanism during infection and how AS may contribute to infection.

## Materials and methods

### *Genome sequencing and annotation of* M. oryzae *strain KJ201*

Genomic DNA of KJ201 was purified using the blood and cell culture DNA midi kit (Qiagen, Germany). Sequencing was performed using a whole-genome shotgun strategy with Illumina Genome Analyser IIx (Beijing Genome Institute, Shenzhen, China). The genome was assembled using SOAPdenovo 1.05 [65] and deposited to NCBI (Accession No. ANSL00000000). The assembled genome was annotated using the Maker 2.31.8 pipeline [66]. We used the built-in PROMER package in MUMMER v3.23 to align the 70–15 and KJ201 genome sequences.

### Transcriptome data analysis

We downloaded published transcriptome datasets generated using KJ201 [41] from NCBI (Accession Nos. SRR8259727 to SRR8259732). The adapter sequences were removed via Cutadapt-1.8.1 using truseq universal sequences [67]. Truncated reads with low quality at the 3’ end and the length shorter than 20 bp were also removed. Only the pair mapped sequences to the genome were used to eliminate those with false-positive splicing junctions (single pair and discordantly mapped reads were removed, using the option of – no-mixed – no-discordant). The remaining reads were aligned to the KJ201 genome using HISAT2-2.1.0 [68].

### Identification of the transcripts generated via AS

A two-step process was used for their identification (Supplementary Figure S2). Initially, consensus sequences of individual isoforms were identified using reference annotation-based transcript assembly processed using StringTie 1.3.5 (the minimum isoform abundance of 0.01) [69]. Information for isoforms, including the type of isoforms, was recorded as General Feature Format (GFF) using ASTALAVISTA 4.0 [70] and eventGenerator embedded in SUPPA2 [71]. Subsequently, the expression value of each gene was calculated using the default settings of Cufflinks 2.2.1 [72]. The transcripts with FPKM value less than one and fused to neighbouring genes were eliminated.

### Identification of translated isoforms and their predicted functions

The sequences of all annotated forms and isoforms of transcripts were retrieved using gffread v0.9.8c from constructed GFF [73]. The transcripts with low coding potential were filtered out using CPC 2.0 [74]. The isoforms with coding potential were classified into NMD candidate transcript (PTC+) and potential protein encoding transcript (PTC-). The PTC position, which is defined as the stop codon within 50 nt upstream of the last exon junction or in intronic sequences, was calculated to eliminate potential NMD candidates. After removing the NMD candidates, the remaining isoforms were translated using getORF (translation from start to stop codon) in EMBOSS v 6.0.0 [75]. There are several potential translation models that did not start at the start codon of annotated form [76]. Because we could not determine the authentic translation start site from RNA-seq data and leaky scanning translation could be possible [77], we predicted proteins using both the annotated start codon and the novel start codon from the longest ORF. First, we collected the proteins that started from the same start codon of annotated gene. Second, we used the longest ORF in transcripts without non-overlapping upstream ORF to eliminate the isoforms covering multiple genes. These two predicted proteomes were merged for subsequent analyses. Putative functional domains of the predicted proteins were identified using Pfam information of InterProScan v68 [78]. Functional category of each gene product was predicted using GO term information of InterProScan v68 [78]. The enrichment of GO term analysis was performed using the web-based tool WEGO 2.0 (http://wego. genomics.org.cn) [79]. The protein sequences derived from predicted isoforms were used to query the proteins archived in PHI-base using BLASTP-2.2.26.

### Gene family analysis

The splicing regulatory factors (SR proteins and hnRNP) were identified by searching the gene products containing RNA recognition motif (RRM) using InterProScan v68 [78]. Among these proteins, we identified those carrying RS domain (SR protein) and G rich motif (hnRNP) using the following rules: N-terminal RRMs and downstream RS domain of at least 50 amino acids with >40% R[S/D] sequence and G-rich motif characterized by consecutive repeats [7, 80]. Putative TFs were identified using the Fungal Transcription Factor Database (FTFD) pipeline [81]. Putative kinases were identified using Hidden Markov Models of the protein sequences from Superfamily (v1.75), Kinomer, and Microbial Kinome [82–84]. Secreted proteins were identified using the Fungal Secretome Database [85]. We collected all signal peptide-containing proteins and assessed their likelihood of secretion using SignalP 5.0b [86] and TMHMM-2.0c [87]. The longest ORF sequences without upstream uORF were used for this analysis. Identification of putative effectors, including those with the size of ≤300 amino acids and excluding putative enzymes, was performed as previously described [88].

### Validation of the production of AS transcripts and their translation

We validated the production of highly expressed isoforms in infected rice plants and also checked their translation. Rice plants were grown in a growth chamber set at 28°C and 80% humidity with 16 h-light/8 h-dark photoperiod. Four-week-old rice cultivar Nakdong was inoculated with KJ201 conidial suspension (5 × 104 conidia/mL in 250 ppm tween20) using a sprayer. The inoculated plants were incubated for 3 and 6 days, including 12 hours in dew chamber. Total RNAs were isolated from infected leaves using the Easy-spin total RNA extraction kit (iNtRON Biotechnology, Korea) according to the manufacturer’s instructions. First-strand cDNA was synthesized from 2 μg of total RNAs using the ImProm-II Reverse Transcription System (Promega) with oligo (dT) primers. Real-time PCR (RT-PCR) reactions were performed using i-star-max II premix (iNtRON Biotechnology, Korea) and primers designed to amplify tested isoforms (Supplementary Table S12). Each reaction consisted of PCR master mix, 25 ng of cDNA, and 15 pmol of each primer. The PCR cycling conditions were 10 min at 94°C followed by 30 cycles of 15s at 94°C and 1 min at 60°C. Poly-Acrylamide Gel Electrophoresis (PAGE) was performed to analyse RT-PCR products.

We analysed the proteomes extracted from infected rice leaves (7 dpi) and mock sample (0 hpi). Protein digestion was conducted using a filter-aided sample preparation (FASP) approach as previously reported [89]. The collected peptides were dissolved in solvent-A (2% acetonitrile (ACN) in water (v/v) with 0.1% formic acid) and separated via reversed-phase chromatography using a UHPLC Dionex UltiMate ® 3000 (Thermo Fisher Scientific, MA, USA). The LC analytical gradient was run 90 min at 2% to 35% solvent B (100% ACN and 0.1% formic acid). Liquid chromatography-tandem mass spectrometry (LC-MS/MS) was performed with an electrospray ionization using QExactive™ Hybrid Quadrupole-Orbitrap High-Resolution Mass Spectrometer (Thermo Fisher Scientific, MA, USA). The peptides were electro-sprayed through a coated silica tip (Scientific Instrument Service, NJ, USA) at an ion spray voltage of 2000 eV, and MS spectra were collected at a resolution of 70,000 (200 m/z) in a mass range of 350-1650 m/z. The MaxQuant (version 1.5.3.30) was used for database searching [90,91]. All three technical replicates were merged to profile the proteins

## Supplementary Material

Supplemental Material

## Data Availability

The assembly data of *M. oryzae* KJ201 genome was deposited GenBank under the accession number of ANSL00000000. The version described in this article is ANSL00000000 (https://www.ncbi.nlm.nih.gov/nuccore/ANSL00000000.2). The proteome data deposited in PRIDE (Submission ID: PXD026316). The associated Bioproject number is PRJNA179498.
